# Exotic vortex lattices in a rotating binary dipolar Bose-Einstein condensate

**DOI:** 10.1038/srep19380

**Published:** 2016-01-18

**Authors:** Xiao-Fei Zhang, Lin Wen, Cai-Qing Dai, Rui-Fang Dong, Hai-Feng Jiang, Hong Chang, Shou-Gang Zhang

**Affiliations:** 1 Key Laboratory of Time and Frequency Primary Standards, National Time Service Center, Chinese Academy of Sciences, Xi’an 710600, People’s Republic of China; 2 College of Physics and Electronic Engineering, Chongqing Normal University, Chongqing 400047, People’s Republic of China; 3 School of Science, Zhejiang Agriculture and Forestry University, Lin’an, Zhejiang 311300, People’s Republic of China

## Abstract

In the last decade, considerable advances have been made in the investigation of dipolar quantum gases. Previous theoretical investigations of a rotating binary dipolar Bose-Einstein condensate, where only one component possesses dipole moment, were mainly focused on two special orientations of the dipoles: perpendicular or parallel to the plane of motion. Here we study the ground-state and rotational properties of such a system for an arbitrary orientation of the dipoles. We demonstrate the ground-state vortex structures depend strongly on the relative strength between dipolar and contact interactions and the rotation frequency, as well as on the orientation of the dipoles. In the absence of rotation, the tunable dipolar interaction can be used to induce the squeezing or expansion of the cloud, and to derive the phase transition between phase coexistence and separation. Under finite rotation, the system is found to exhibit exotic ground-state vortex configurations, such as kernel-shell, vortex necklace, and compensating stripe vortex structures. We also check the validity of the Feynman relation, and find no significant deviations from it. The obtained results open up alternate ways for the quantum control of dipolar quantum gases.

Owing to the high degree of control over most of the system parameters, ultracold or even degenerate atomic quantum gases offer us a unique platform for the study of macroscopic quantum phenomena. In a cold dilute atomic gas, atoms predominantly interact with each other through the short-rang van der Waals interaction, which can be described in terms of elastic *s*-wave scattering length and is regarded as the origin of most phenomena that have been observed in dilute Bose-Einstein condensate (BEC)^1^,^2^. However, recent experiments on atoms with large magnetic moments and polar molecules, such as ^52^Cr (6*μ*_*B*_), ^168^Er (7*μ*_*B*_), and ^164^Dy (10*μ*_*B*_) with *μ*_*B*_ being the Bohr magneton, are attracting major interest to the physics of dipolar quantum gases in which dipole-dipole interaction (DDI) is important^3^,^4^,^5^,^6^,^7^,^8^,^9^,^10^.

Due to the long-range and anisotropic nature of the DDI, ultracold atoms with a magnetic (or electric) dipole moment exhibit a variety of intriguing phenomena, such as the anisotropic deformation and excitation^11^,^12^, *d*-wave collapse and expansion^13^,^14^, supersolidity^15^, roton spectrum^16^,^17^,^18^, and also the rotating properties^19^,^20^,^21^,^22^,^23^. Notably, attribute to the Feshbach resonance, one can tune the *s*-wave scattering length between −∞ and +∞, leading to the realization of a purely dipolar gas. Furthermore, it is experimental realization that the DDI can be reduced its natural value, including to negative values, by external field rotation, which leads to a large parameter space^24^.

One of the characteristic properties of superfluid is that they respond to rotation by forming quantized vortices, which is a universal feature of macroscopic quantum systems with spontaneously broken U(1) symmetry. Vortex physics becomes much more diverse in dipolar gases than in typical short-range interacting ones, due to its long-range and anisotropic^25^,^26^,^27^,^28^,^29^. For a single-component dipolar condensate, the critical rotation frequency, at which a vortex state becomes energetically favorable over the vortex-free ground state was calculated in^30^; and the rotational properties and vortex lattices were studied in^31^,^32^, in which the triangular, square, stripe, and bubble phases were obtained. Later, vortex structures with a relatively small number of vortices in a three-dimensional trapped geometry were considered in^33^, the ground states of rotating condensates with DDI beyond the weak interaction limit was studied in^34^.

So far, most of the previous studies (including the aforementioned works) on dipolar BEC have been restricted to single- or multi-component system, where each component has a dipole moment. Very recently, the ground-state and rotational properties of a binary dipolar gas, wherein only one component possesses magnetic dipole moment, have drawn considerable attentions^35^,^36^,^37^,^38^,^39^. Typically, the half-quantum vortex chain phase and a half-quantum vortex molecule in such a binary (or two-component) dipolar gas with harmonic potential was studied in^36^, and the phase separation in the quasi-one- and quasi-two-dimensional regime was investigated in^37^. More importantly, the vortex competition in a rotating binary dipolar BEC was studied in^38^. However, these works focus on two special orientations of the dipoles: perpendicular or parallel to the plane of motion. To our best of knowledge, there has little work on such a binary system for an arbitrary orientation of the dipoles with respect to the plane of motion.

Motivated by such rich physics and to fill up this gap, it is of particular interest to extend the situation to an arbitrary orientation of the dipoles with respect to the plane of motion, which is what we attempt to do in this work. Our results show that the ground-state phases and vortex structures of such a system depend strongly on the relative strength between the dipolar and contact interactions, and the rotation frequency, as well as on the orientation of the dipoles. The inclusion of DDI introduces another “switch”, which can be used not only to obtain the desired ground-state phase, but also to control the number of vortices and its related vortex structures, even for a *non-dipolar* condensate.

## Results

### Mean-field model

Our studies are restricted to the zero-temperature mean-field regime, where the ground-state and dynamics of a binary dipolar BEC are described in terms of two complex-value order-parameter *ψ*_1_ and *ψ*_2_. We consider a binary BEC confined in a quasi-two-dimensional (Q2D) plane, with the polarization axis being at angle Θ to the *x* axis in the *x*-*z* plane. The Q2D regime is obtained by assuming a sufficiently strong trapping potential in the axial direction *z*, thus the *z* dependence of *ψ*_1_ and *ψ*_2_ can be factored out and all the relevant dynamics occur only in *x* and *y* directions. Hence we arrive at the effective Q2D nonlocal GP equations for the order parameters of a binary dipolar BEC^40^,^41^,^42^,


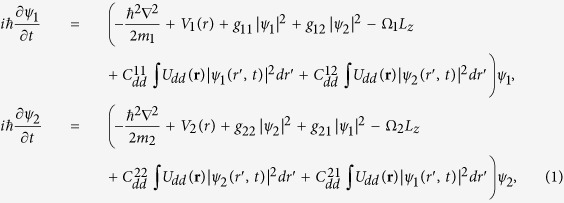


where *ψ*_*i*_ is the condensate wave function, *m*_*i*_ is the atomic mass, Ω_*i*_ is the effective rotation frequency of component *i*, and *L*_*z*_ = −*iħ*(*x*∂_*y*_ − *y*∂_*x*_) is the *z* component of the orbital angular momentum operator. The Q2D external potential can be written as 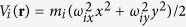. For the isotropic short-range contact interactions, 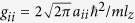 and 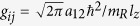 with 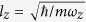 and *m*_*R*_ = *m*_1_*m*_2_/(*m*_1_ + *m*_2_) being the oscillator length in the *z* direction and the reduced mass, describe the intra- and inter-component interactions, respectively. To simplify the numerical analysis and highlight the effects of DDI, it is convenient to reduce the number of free parameters in the problem. Thus, we specialize to the “balanced” case where *ω*_1*x*_ = *ω*_2*x*_ = *ω*_1*y*_ = *ω*_2*y*_ = *ω*, *m*_1_ = *m*_2_ = 2*m*_*R*_ ≡ *m*, *N*_1_ = *N*_2_ = *N*, Ω_1_ = Ω_2_ = Ω, and consider only the fixed repulsive short-rang contact interactions, *i.e.*, *g*_11_ = *g*_22_ = *g*_21_ = *g*_12_ = *g* = 55^43^,^44^, which describe the ground-state properties of the non-dipolar system quite well. Here we want to note that the *effective* contact interactions between atoms can be controlled by modifying atomic collisions (achieved by magnetically tuning the Feshbach resonances), or the atom number *N*. In addition, we assume that component 2 is nondipolar, leading to 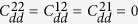 and 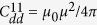 
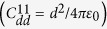
 for magnetic dipoles (electric dipoles), where *μ*_0_ and *μ* are the magnetic permeability of vacuum and the magnetic dipole moment, respectively. For dipolar component 1, we assume all the dipoles are aligned to some external field, which is in the *x*-*z* plane, and forms an angle Θ with the *x*-axis^26^.

For the dipolar component 1, the most important quantity (dimensionless) is the relative strength between the dipolar and contact interactions, *ε*_*dd*_ = *μ*_0_*μ*^2^*m*/12*πħ*^2^*a*_*s*_, which determines the physical properties of such a component. Due to the unprecedented level of experimental control of the strengths of DDI and contact interactions, in principle, such a parameter can be tuned from positive to negative values^5^,^24^. For *ε*_*dd*_ < 1, the short-range part of the interparticle interaction dominates and DDI provides only corrections, which corresponds to a stable BEC. However, large values of *ε*_*dd*_ may make the system unstable against collapse for small values of the angle Θ, due to the anisotropic nature of the DDI.

Now we consider the ground-state and rotational properties of such a system as a function of tunable DDI and its orientation, and of the rotation frequency, and then propose how to obtained the desirable ground-state by synchronizing the DDI and rotation frequency in future experiments.

### Effect of DDI and its orientation on the ground-state configuration

Let us first investigate the ground-state structure of the system as a function of the dipolar strength, and of the dipoles of the atoms with respect to the plane of motion, for fixed contact interactions. To highlight the effects DDI, the system is first set as rest without rotation. It is well-known that for the nonrotational case, the ground-state density distributions of the system depend sensitively on the relative strength between inter- and intra-component contact interactions, showing phase coexistence or phase separation. However, the presence of the tunable dipolar interactions in component 1, worked as another effective *switch*, is found to give rise to novel density distributions.

**Figure 1:**
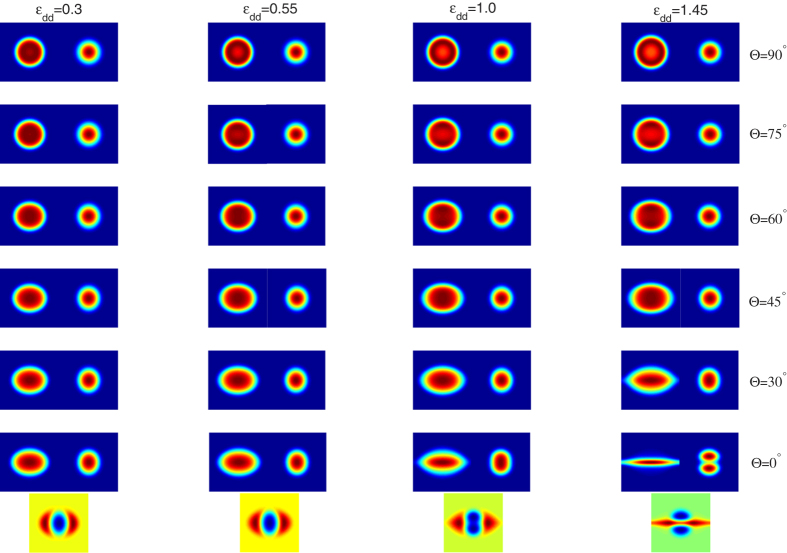
The two-dimensional atom density profiles for each component as a function of the orientation of the dipoles for the nonrotational case. The polarization axis being at angle Θ = 90°, 75°, 60°, 45°, 30°, 0°. The relative strength between the dipolar and contact interactions are set as *ε*_*dd*_ = 0.3, 0.55, 1.0, 1.45. Here the system is set as rest with Ω = 0, and all the contact interactions are fixed at *g* = 55. Note that for each plot, the left (right) one is the density of the dipolar (scalar) component, and the last row denotes the density difference of such two components for Θ = 0°. The scale of each figure is 6.0 × 6.0 in dimensionless unit.

Figure 1 exhibits the typical density distributions of the system for Θ = 90°, 75°, 60°, 45°, 30°, 0°, and for *ε*_*dd*_ = 0.3, 0.55, 1.0, 1.45. First, it is clear from Fig. 1 that such density distributions depend sensitively on both the strength of DDI and the orientation of the dipoles. When the orientation of the dipoles is aligned along the *x*-axis (Θ = 0°), we observe the well-known self-induced squeezing of the cloud along the *y*-axis direction, as shown in the last row of Fig. 1. We note that the same self-induced squeezing was also observed in a rotating single-component dipolar BEC with anisotropic harmonic potential^26^,^45^. Actually, this result can be explained as follows. On one hand, the dipolar interaction, which is attractive in the *x*-axis but repulsive in the *y*-axis, leads to an *effectively* anisotropic effect. On the other hand, the presence of repulsive inter-component interaction between the dipolar and scalar components, together with the anisotropic DDI, result in the squeezing of the dipolar component and the spatial phase separation of such two components.

Upon increasing Θ slowly to 30° and 45°, one find a similar, but less pronounced, change of the density profile, as in these cases the attractive part of the dipolar interaction becomes less important. Further increases of Θ, such as Θ = 60°, the situation is changed. In this case, the repulsive and the attractive parts of the dipole interaction become comparable, leading to a nearly isotropic expansion of the cloud along both the *x*- and *y*-axis. When the orientation of the dipoles exceeds Θ = 75°, the behavior of the system changes, the repulsive part of the dipolar interaction becomes dominant, leading to a net *effective* repulsion. As expected, this effect is maximal when the dipoles are polarized along the *z* axis with Θ = 90°. In this special case, the dipolar interaction is purely repulsive and isotropic, leads to an isotropic expansion of the cloud.

**Figure 2:**
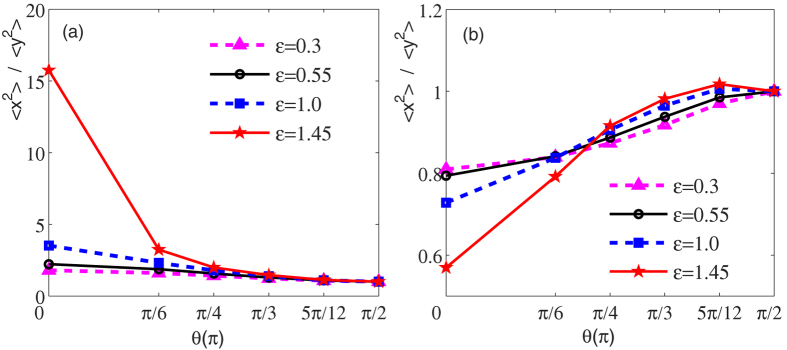
The profiles of the dimensionless parameter 〈*x*^2^〉/〈*y*^2^〉 for the density distributions illustrated inFig. 1. (**a**) Dipolar component 1; (**b**) Non-dipolar component 2.

To give a more clear explanation of the above results, we introduce a dimensionless parameter 〈*x*^2^〉/〈*y*^2^〉 to describe the self-induced squeezing. Figure 2 shows the profiles of such parameter for the density distributions illustrated in Fig. 1, where the left (right) one is for the dipolar (non-dipolar) component. It is easy to see that with an increase of the relative strength between the dipolar and contact interactions, the self-induced squeezing of the cloud along the *y*-axis direction performs more obviously for dipolar component, while the picture is reversed for non-dipolar one. In addition, when the dipoles are polarized along the *z* axis, we always get an isotropic cloud, regardless of its value.

Hence we conclude that when the system is set as rest without rotation, not only the strength of DDI but also its orientation can be regarded as two “degrees of freedom”, which can be used to induce the squeezing and isotropic (anisotropic) expansion of the cloud, and to derive the phase transition between phase coexistence and phase separation. Moreover, the inter-component parameter *g*_12_ significantly affects the atom density distributions, even for a trapped binary dipolar condensate.

### The combined effect of rotation frequency and DDI on vortex configuration

It is also of interest to investigate the rotational properties of the system as a function of the rotation frequency, which manifest itself as the presence of quantized vortices. Thus we now turn our attention to the effect of rotation frequency on the ground-state vortex configuration of such a system. It is found that the vortex lattice will form with a structure strongly dependent on both the tunable DDI and the rotation frequency.

For a relatively slow rotation, we find a similar behavior for the density distribution of such two components as the nonrotational case. The only difference is that the phase separation occurs at a relatively small *ε*_*dd*_ within the range that we have considered. Take Ω = 0.3 as an example, in this case the system shows phase separation at a relatively small value *ε*_*dd*_ = 1. However, our results below show that further increasing the rotation frequency Ω will induce new nucleated vortices in both dipolar and scalar components.

**Figure 3:**
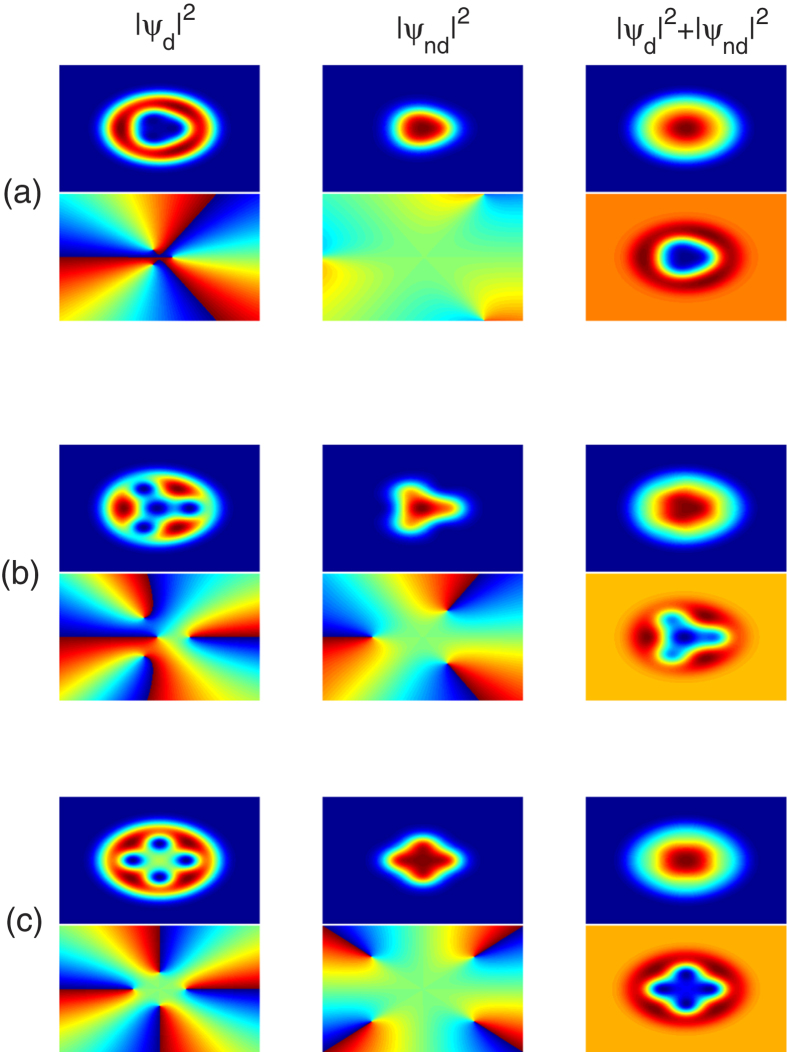
Kernel-shell structure of a rotating binary dipolar BEC. The two-dimensional atom density and phase profiles of the system as a function of the relative strength between dipolar and contact interactions, for *ε*_*dd*_ = 0.3, 0.55, 1.0, corresponding to (**a**–**c**), respectively, showing three typical vortex structures. Here the dipolar atoms in the first component 1 are polarized perpendicular to *x*-axis, *i.e.*, Θ = 90°, the rotation frequency is fixed to Ω = 0.5, and all the contact interactions are fixed at *g* = 55. The corresponding phase distributions for *ψ*_*d*_ and *ψ*_*nd*_, and the density difference of such two components are shown in the lower panel for each plot, and the scale of each figure is 6.0 × 6.0 in dimensionless unit.

At first, we consider the situation where the dipolar atoms in the first component 1 are polarized perpendicular to the plane of motion, *i.e.*, Θ = 90°. Figure 3 plots the typical ground-state density and phase distributions of the system for a mediate rotation frequency Ω = 0.5, but for varied *ε*_*dd*_, showing three typical vortex structures. From these images, we observe that in this case the system is always in phase separation. Interestingly, the density distribution of such a system exhibits a “kernel-shell” structure, in which the dipolar component occupy the outside regime and forms a shell-like structure, while the scalar component occupy the center regime with a structure strongly dependent on the strength of the DDI. It should be mentioned that the “kernel-shell” structure has been previously observed in a rotating two-component dipolar BEC, where dipolar component mainly occupy the center regime^38^. The explanation of the above role exchange lies in the different orientations of the dipoles. For Θ = 90° considered in the present study, the DDI is purely repulsive and isotropic, leads to 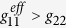. Thus, the dipolar atoms which have kinetic energies high enough that they can overcome the trapping potential and occupy the outside regime. Finally, while the system considered here is nominally similar with the one in^38^, there also exist subtle differences. For example, in the latter case, the strength of DDI is fixed and the variation of *g*′ leads to a variation of inter-component interaction, whereas in the present study the inter-component interaction *g*_12_ is fixed. In addition, we assuming the dipoles are aligned along some arbitrary and tunable direction, whereas only vertical and parallel situation were considered in the latter case.

Further information can be extracted from the phase distribution and the density difference of such two components [see the lower panel for each plot of Fig. 3]. Remarkably, such phase profiles reveal that with an increase of the strength of DDI, more and more vortices enter the condensate and form a vortex lattice, which is in a sense reminiscent of the cases in^26^. Thus, the three vortices present for *ε*_*dd*_ = 0.3 get increased to four for *ε*_*dd*_ = 0.55, 1.0, where different vortex structures are formed to lower the total energy of the system.

**Figure 4:**
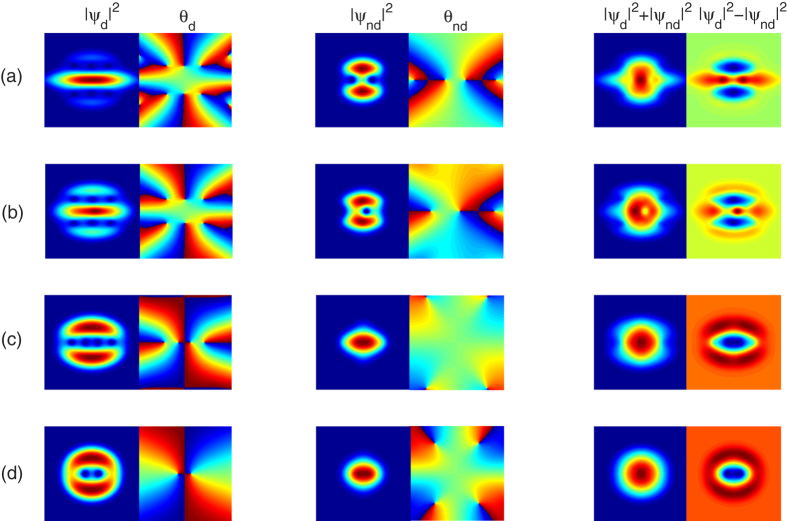
Self-induced squeezing to Kernel-shell transition induced by the orientation of the dipoles. The two-dimensional atom density and phase profiles as a function of the orientation of the dipoles, for Θ = 30°, 45°, 60°, 75°, corresponding to (**a**–**d**), respectively. Here the rotation frequency is fixed to Ω = 0.5, the relative strength between the dipolar and contact interactions is fixed to *ε*_*dd*_ = 1.0, and all the contact interactions are fixed at *g* = 55. The scale of each figure is 6.0 × 6.0 in dimensionless unit.

Next we study the effects of the orientation of the dipoles on the rotational properties of the system. In our numerical simulations, it is found that for fixed rotation frequency and the strength of the dipolar interaction, the ground-state vortex structure of the system, as a function of the orientation of the dipoles, shows similar behavior. Figure 4 shows the ground-state density and phase distributions as a function of the orientation of the dipoles for Θ = 30°, 45°, 60°, 75°, for a fixed rotation frequency Ω = 0.5, and for a fixed relative strength between the dipolar and contact interactions *ε*_*dd*_ = 1.0. For Θ = 30°, again we find the self-induced squeezing of dipolar component. However, compared with the nonrotational case, three vortex lines (two for dipolar component and one for scalar one) forms due to the presence of rotation. As the orientation of the dipoles increases, stripe pattern appears, as shown in Fig. 4(b) for Θ = 45°, and such stripe pattern will be more and more obvious with increasing the rotation frequency. Further increases of Θ, such as Θ = 60°, 75° for Fig. 4(c,d), the density profiles of the system gradually develop to the “kernel-shell” structure [see Fig. 3(c) for Θ = 90°].

**Figure 5:**
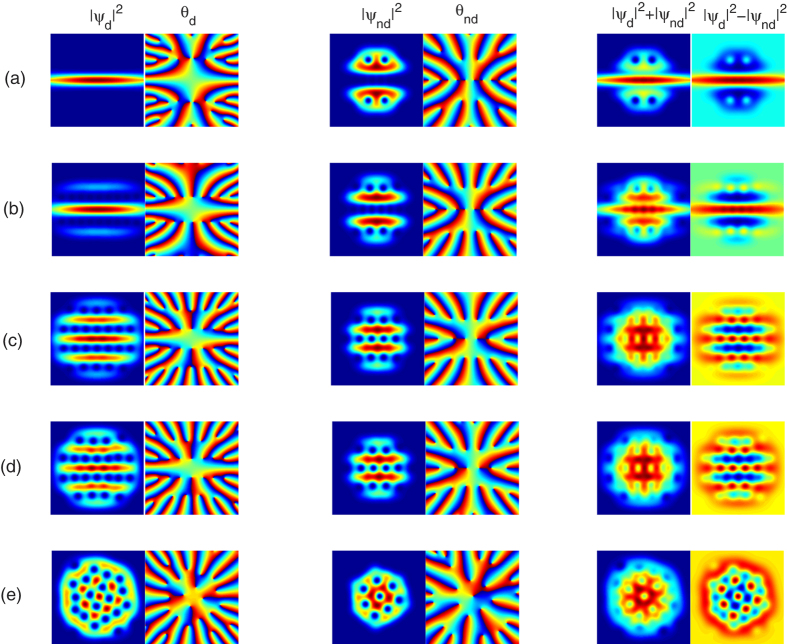
Self-induced squeezing-stripe pattern-vortex necklace transition induced by the orientation of the dipoles. The two-dimensional atom density profiles as a function of the orientation of the dipoles, for Θ = 0°, 30°, 60°, 75°, 90°, corresponding to (**a**–**e**), respectively. Here the rotation frequency is fixed to Ω = 0.9, the relative strength between the dipolar and contact interactions is fixed to *ε*_*dd*_ = 0.9, and all the contact interactions are fixed at *g* = 55. The scale of each figure is 6.0 × 6.0 in dimensionless unit.

Finally, we illustrate the ground-state vortex structure of the system for a rapid rotation Ω = 0.9. In this case, a rapid rotation will generate a dense array of vortices, which are arranged spontaneously as a vortex lattice to minimize the total energy of the system^22^. Figure 5 presents the ground-state density and phase distributions as a function of the orientation of the dipoles for Θ = 0°, 30°, 60°, 75°, 90°, for a fixed rotation frequency Ω = 0.9 and for a fixed relative strength between the dipolar and contact interactions *ε*_*dd*_ = 0.9. At Θ = 0°, no regular lattice structure forms for the dipolar component; whereas the vortices (including hidden one) in the scalar component form three vortex lines, where two bend lines present even parity with respect to the central one (which is also an imaging line along the main dipolar condensate), as shown in Fig. 5(a).

As the orientation of the dipoles increases, the ground-state profiles change gradually into an alternatively arranged stripe pattern, where both the dipolar and scalar atoms are arranged into compensating parallel stripes. It is easy to find that the numbers of vortices in both the dipolar and scalar components increases with increasing Θ. Meanwhile, the locations of the vortices of one component are at the region of the density domains of the other component, and the vortices of each component are overlapped in lines and the density forms a stripe pattern, as shown in Fig. 5(b–d) for Θ = 30°, 60°, 75° 46. Actually, as discussed previously, such stripe structure may be viewed as a phenomenon of phase separation, and we attribute this to the anisotropic nature of the DDI^33^,^47^. For the vertical situation, the DDI is purely repulsive, which drastically increases the effectively contact-like interactions, leading to a necklace vortex structure, as shown in Fig. 5(e).

Last but not least, we want to note that while the numerical results presented above are limited to some special values of the system parameters, qualitatively similar behavior is also observed over a range of different parameter sets. Actually, as discussed above, for the non-dipolar condensate, the effective contact interactions between atoms *g* can be controlled by modifying atomic collisions, or the atom number N. For the dipolar component, the most important quantity is the relative strength between the dipolar and contact interactions *ε*_*dd*_. Thus the parameter sets used in our manuscript illustrate well the possible ground states. Furthermore, it is also of interest to investigate the validity of the Feynman relation for the mean vortex density. For a two-component system, it naturally generalizes to


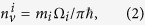


where *m*_1_ and *m*_2_ are the masses of the bosons in the two constituent condensates, and Ω_1_ and Ω_2_ are the angular rates at which the two superfluids are rotating^19^,^22^,^48^,^49^,^50^. For the system considered here, we specialize to the “balanced” case where *m*_1_ = *m*_2_ = *m*, and Ω_1_ = Ω_2_ = Ω, which leads to the same areal density of vortices for such two components, as shown in Fig. 5 for a rapid rotation Ω = 0.9. Here we want to note that, for a mediate rotation frequency, such as Ω = 0.5 shown in Figs 3 and 4, the density of vortices (including the hidden and elusive ghost vortices) of such two components are almost equal to each other^51^.

In light of these results we can better understand the combined effect of rotation and DDI on the ground-state vortex arrangement, and conclude that use of a binary dipolar BEC, wherein only one component possesses dipole moment, is another possible way of creating novel vortex pattern formation without suffering a dipolar collapse^52^,^53^.

## Discussion

In summary, we have investigated the effects induced by the anisotropic nature of the dipolar interaction in a rotating binary dipolar BEC, which consists of both dipolar and scalar bosonic atoms and confined by a quasi-two-dimensional harmonic trapping potential. We have considered the dipole moment to be oriented on the *x*-*z* plane, forming an arbitrary and tunable angle with *x*-axis.

We have analyzed the ground-state and rotational properties of the system for different strength of DDI and its orientation, and for different rotation frequency. For the nonrotational case, our results show that the strength of DDI and the orientation of the dipole moment, worked as two different *switches*, which can be used to quantum control of the ground-state density of such a system. Upon the increases of the rotation frequency, the new nucleated vortices enter each component, and the system exhibits exotic ground-state structure, such as kernel-shell, vortex necklace, and compensating stripe vortex structure, due to the anisotropic characteristic of the dipolar interaction. Finally, we have checked the validity of the Feynman relation of the system and found no significant deviations from it.

One point of particular importance is that our results may shed insight on problems focusing on the interplay between isotropic inter-component and anisotropic dipolar interactions, which has not been studied as thoroughly. To estimate the physical realizability of the described effects, perhaps the most promising system is a binary BEC, which can be realized by selecting two states in the ground hyperfine manifolds of atomic Cr, Dy, or Er, where components 1 and 2 consist of states with spin projections *m*_*J*_ = −*J* and *m*_*J*_ = 0. With regard to the relative strength between the DDI and contact interaction, typically, for ^52^Cr with magnetic dipole moment *d* = 6*μ*_*B*_ and *s*-wave scattering length *a*_*s*_ ≈ 100*a*_0_ (*a*_0_ being the Bohr radius), *ε*_*dd*_ = 0.036^54^. However, in real experiments, the contact interactions can be controlled by tuning the Feshbach resonance, making the DDI dominant.

## Methods

The long-range and nonlocal DDI for component 1 has the form 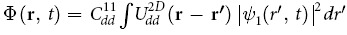 with


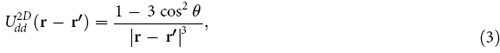


where *θ* is the angel between the polarization direction and the interatom vector **r** − **r′**. To deal with this DDI, it is convenient to work in Fourier space and use the convolution theorem^39^,^55^,^56^,


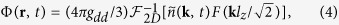


where 
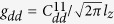
 is the DDI coupling, 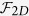 is the 2D Fourier transform operator and 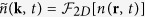. The function *F*(**q**) with 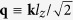 is the *k* space DDI for the Q2D geometry, which consists two parts coming from polarization perpendicular or parallel to the direction of the dipole tilt. That is 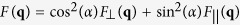 with *α* being the angle between 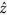 and the polarization vector 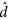. Here 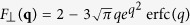, 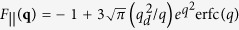, **q**_*d*_ is the wave vector along the direction of the projection of 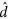 onto the *x*-*y* plane, and erfc is the complementary error function^57^. Here we want to note that if the polarization is perpendicular to the condensate plane, we get 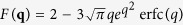, as discussed in previous works^35^,^36^,^58^.

To study the ground-state and rotational properties of the system, we start from proper initial wave-functions and use the split-step method within an imaginary-time propagation approach^59^,^60^,^61^. The lowest-energy states in different parameter space are obtained until the fluctuation in the norm of the wave function becomes smaller than 10^−7^. In the numerically simulations, we work in dimensionless units by introducing the scales characterizing the trapping potential: the length and time are scaled as 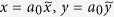, and 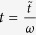, respectively, with 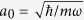 (here the tilde is omitted for simplicity).

## Additional Information

**How to cite this article**: Zhang, X.-F. *et al.* Exotic vortex lattices in a rotating binary dipolar Bose-Einstein condensate. *Sci. Rep.* **6**, 19380; doi: 10.1038/srep19380 (2016).
